# Dietary restriction and other lifespan extending pathways converge at the activation of the downstream effector takeout

**DOI:** 10.18632/aging.100174

**Published:** 2010-07-16

**Authors:** Martina Gáliková, Thomas Flatt

**Affiliations:** Institute of Population Genetics, Department of Biomedical Sciences, University of Veterinary Medicine Vienna, A-1210 Vienna, Austria

Dietary restriction (DR), reduced food uptake without malnutrition, is the most universal intervention known to extend animal lifespan, from invertebrates to mammals [1]. However, despite impressive progress in identifying the key components of the DR pathway, many proximal effectors of DR induced longevity remain unknown to date [1]. One central obstacle in the search for such mechanisms is that DR causes a myriad of transcriptional and physiological changes that are either not - or only indirectly - related to its positive effects on lifespan. For example, DR often increases lifespan at the expense of fertility [1]. In turn, the inability to filter out non-longevity effects of DR might impede the development of therapeutic compounds that mimic DR without causing unwanted side effects. For instance, while manipulation of dietary methionine can uncouple the phenotypic association between DR induced longevity and decreased fertility [2-3], whether and how such pleiotropic effects of DR are functionally separable at the molecular level is currently not understood. In this May issue of AGING, Bauer and colleagues now make a major step towards identifying those downstream effectors of DR that specifically affect longevity [4].

**Figure 1.**
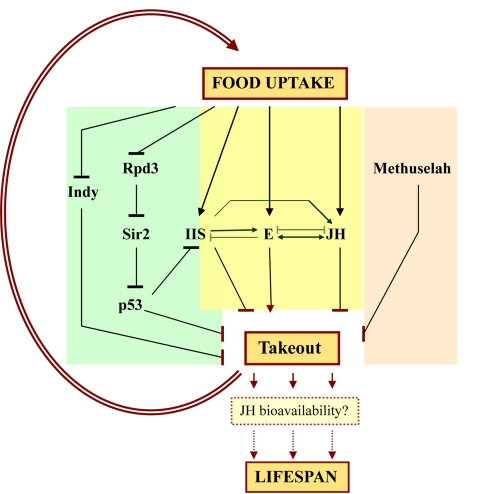
The study by Bauer et al. places *takeout *(*to*) in a central, hub-like regulatory position in the lifespan regulatory network. *to* might modulate lifespan via different avenues: as a component of the DR pathway (e.g., *Indy*, *Rpd3*, *Sir2*, *p53*) and by regulating food uptake; by receiving signals from nutritional signaling pathways such as IIS (e.g., *chico*); by its potential involvement in lipophilic hormone signaling and metabolism (ecdysone [E], juvenile hormone [JH]), for example by regulating JH bioavailability; and by unknown interactions with G protein-coupled receptor signaling (*methuselah*).

To find the genetic key players that mediate lifespan extension upon DR, Bauer et al. used comparative whole genome expression profiling by searching for genes whose expression patterns overlap between DR treatment and two signaling states that are functionally related to DR but that do not affect fertility, activation of *Sir2* and inactivation of *p53* in the fly brain. Using this approach the authors identified a small set of shared transcriptional changes in 21 genes (20 genes up- and one downregulated) that are involved in chromatin structure or maintenance, circadian rhythm, neural activity, detoxification and chaperone activity, muscle maintenance, immunity, growth factor activity, and nutrient storage. To further narrow this list, Bauer et al. performed qPCR for these genes in four additional long-lived mutants (*Indy* and *Rpd3*, both implicated in DR; the insulin receptor substrate *chico*; and the G protein-coupled receptor *methuselah*). Remarkably, the authors found a single gene, *takeout *(*to*), to be robustly upregulated in all seven longevity promoting conditions examined. Next, to confirm that *to *is a specific effector downstream of DR and other longevity pathways, Bauer et al. overexpressed *to *in adult neurons, pericerebral fat body and abdominal fat tissue and found that these manipulations extend lifespan in both females and males. Moreover, long-lived flies overexpressing *to* exhibited upregulation of nine genes (out of the 19 remaining genes identified above) that were also induced by DR, *Sir2* activation, and *p53* inactivation. Taken together, these results clearly identify *to* as a central target downstream of DR and other longevity pathways (Figure 1).

But what is so special about *to* that might explain its hub-like position downstream of DR and other longevity pathways? Although the detailed molecular function of *to* remains unknown, there are several interesting hints that allow us to speculate about the mechanisms whereby *to* might regulate lifespan. The perhaps most obvious connection between *to* and longevity is its involvement in the circadian regulation of food uptake [5-6]. Expression of *to* is induced by starvation, which is blocked in arrhythmic central clock mutants, and *to* mutants die rapidly in response to starvation [5]. Given the role of *to* in feeding behavior, it would thus be natural to ask in future work whether *to* is strictly required for the lifespan response to DR, a food condition that is much milder than starvation, and how in general activation or inactivation of *to* modulates lifespan across a range of different diets. In addition, it would be interesting to know how manipulating *to* activity under different food conditions affects food intake and nutrient metabolism. Such future experiments will likely clarify the physiological role of *to* in affecting diet induced changes in lifespan.

Recent work on two insect hormones provides another potentially significant connection between *to* and longevity and points towards an involvement of *to* in the endocrine regulation of lifespan. In a microarray study on female fly ovaries, Terashima and Bownes [7] confirmed that *to* is starvation inducible and found that *to* expression is oppositely regulated by two lipophilic hormones, ecdysone and juvenile hormone. Ecdysone (E) and juvenile hormone (JH) are endocrine master regulators that often interact to regulate many aspects of insect development and physiology; both hormones are known to respond to nutritional status and insulin/IGF-1 like signaling (IIS) [8-10]. That *to*is transcriptionally regulated by two nutrient responsive hormones downstream of IIS is particularly interesting in view of the fact that both E and JH affect insect lifespan [9,11-13]. *Drosophila*mutants of the ecdysone receptor *EcR* are long-lived, and surgical or genetical ablation of the gland producing JH extends lifespan in grasshoppers, butterflies, and *Drosophila *[9,11-13]. Remarkably, sequence analysis with an iterated BLAST search has identified the TO protein as a putative JH binding protein [5], and it thus tempting to speculate that *to* might regulate lifespan by modulating JH bioavailability. In this context, it will be of major interest to learn in future experiments whether and how changes in *to* activity impact JH signaling and metabolism and whether *to* overexpression is still able to extend lifespan in JH deficient flies [13].

Whatever the potential mechanisms to be discovered, the authors' observation that *to* is induced by DR and four DR related genetic manipulations (*Sir2*,* p53*,* Indy*,* Rpd3*) provides compelling evidence that *to* is part of the DR longevity pathway. While it is clear that only detailed epistasis analyses can determine the exact position of *to* within distinct or overlapping longevity pathways, the study by Bauer et al. elegantly illustrates the power of using comparative genome-wide gene expression profiling for identifying central downstream effectors of longevity pathways.
